# Impact of JAK Inhibitors in Pediatric Patients with STAT1 Gain of Function (GOF) Mutations—10 Children and Review of the Literature

**DOI:** 10.1007/s10875-022-01257-x

**Published:** 2022-04-29

**Authors:** Angela Deyà-Martínez, Jaques G. Rivière, Pérsio Roxo-Junior, Jan Ramakers, Markéta Bloomfield, Paloma Guisado Hernandez, Pilar Blanco Lobo, Soraya Regina Abu Jamra, Ana Esteve-Sole, Veronika Kanderova, Ana García-García, Mireia Lopez-Corbeto, Natalia Martinez Pomar, Andrea Martín-Nalda, Laia Alsina, Olaf Neth, Peter Olbrich

**Affiliations:** 1 Study Group for Immune Dysfunction Diseases in Children (GEMDIP), Institut de Recerca Sant Joan de Déu, Barcelona, Spain; 2 Clinical Immunology and Primary Immunodeficiencies Unit, Pediatric Allergy and Clinical Immunology Department, Hospital Sant Joan de Déu, Barcelona, Spain; 3 Clinical Immunology Program Hospital, Sant Joan de Déu-Hospital Clínic Barcelona, Barcelona, Spain; 4 Infection in Immunocompromised Pediatric Patients Research Group, Vall d’Hebron Institut de Recerca (VHIR), Hospital Universitari Vall d’Hebron, Vall d’Hebron Barcelona Hospital Campus, Barcelona, Catalonia Spain; 5 Pediatric Infectious Diseases and Immunodeficiencies Unit, Hospital Universitari Vall d’Hebron, Vall d’Hebron Barcelona Hospital Campus, Barcelona, Catalonia Spain; 6 Jeffrey Modell Diagnostic and Research Center for Primary Immunodeficiencies, Barcelona, Spain; 7 Division of Immunology and Allergy, Dept of Pediatrics, Ribeirão Preto Medical School, University of São Paulo, Ribeirão Preto, Brazil; 8 Department of Pediatrics, Hospital Universitari Son Espases, Palma, Spain; 9 Multidisciplinary Group for Research in Peadiatrics, Hospital Universitari Son Espases, Balearic Islands Health Research Institute (IdISBa), Palma, Spain; 10 Department of Immunology, 2nd Faculty of Medicine, Charles University and University Hospital Motol, Prague, Czech Republic; 11 Department of Pediatrics, 1st Faculty of Medicine, Charles University in Prague, Thomayer University Hospital, Prague, Czech Republic; 12 Laboratorio de Alteraciones Congénitas de La Inmunidad, Instituto de Biomedicina de Sevilla (IBiS), Laboratorio 205, Seville, Spain; 13 Inborn Errors of Immunity Group, Laboratory 205, Instituto de Biomedicina de Sevilla (IBiS), Seville, Spain; 14 Pediatric Rheumatology Unit, Rheumatology Department, Hospital Universitari Vall d’Hebron, Vall d’Hebron Barcelona Hospital Campus, Barcelona, Catalonia Spain; 15 Immunology Department, Hospital Universitari Son Espases, Palma, Spain; 16 Human Immunopathology Research Laboratory, Institut d’Investigació Sanitària de Les Illes Balears (IdISBa), Palma, Spain; 17 Universitat de Barcelona, Barcelona, Spain; 18 Pediatric Infectious Diseases, Rheumatology and Immunology Unit, Hospital Universitario Virgen del Rocio, Sevilla, Spain; 19 Departamento de Farmacología, Pediatría y Radiología. Facultad de Medicina, Universidad de Sevilla, Seville, Spain

**Keywords:** Primary immunodeficiency disease, Inborn errors of immunity, Pediatrics, Children, JAK-STAT pathway, Chronic mucocutaneous candidiasis, Ruxolitinib, Baricitinib, STAT1 GOF, JAK inhibitors

## Abstract

**Introduction:**

Since the first description of gain of function (GOF) mutations in signal transducer and activator of transcription (STAT) 1, more than 300 patients have been described with a broad clinical phenotype including infections and severe immune dysregulation. Whilst Jak inhibitors (JAKinibs) have demonstrated benefits in several reported cases, their indications, dosing, and monitoring remain to be established.

**Methods:**

A retrospective, multicenter study recruiting pediatric patients with STAT1 GOF under JAKinib treatment was performed and, when applicable, compared with the available reports from the literature.

**Results:**

Ten children (median age 8.5 years (3–18), receiving JAKinibs (ruxolitinib (*n* = 9) and baricitinib (*n* = 1)) with a median follow-up of 18 months (2–42) from 6 inborn errors of immunity (IEI) reference centers were included. Clinical profile and JAKinib indications in our series were similar to the previously published 14 pediatric patients. 9/10 (our cohort) and 14/14 patients (previous reports) showed partial or complete responses. The median immune deficiency and dysregulation activity scores were 15.99 (5.2–40) pre and 7.55 (3–14.1) under therapy (*p* = 0.0078). Infection, considered a likely adverse event of JAKinib therapy, was observed in 1/10 patients; JAKinibs were stopped in 3/10 children, due to hepatotoxicity, pre-HSCT, and absence of response.

**Conclusions:**

Our study supports the potentially beneficial use of JAKinibs in patients with STAT1 GOF, in line with previously published data. However, consensus regarding their indications and timing, dosing, treatment duration, and monitoring, as well as defining biomarkers to monitor clinical and immunological responses, remains to be determined, in form of international prospective multicenter studies using established IEI registries.

**Supplementary Information:**

The online version contains supplementary material available at 10.1007/s10875-022-01257-x.

## Introduction

Since its first description in 2011 [1, 2], gain of function (GOF) mutations in signal transducer and activator of transcription (STAT) 1 have been identified in more than 300 patients worldwide. Most mutations are localized in the Src homology 2 (SH2) or DNA-binding domains [3]. STAT1 is mainly activated via the binding of type I, II, and III interferons to their respective cytokine receptors, resulting in JAK1, JAK2, and JAK3 activation and phosphorylation, followed by the recruitment of STAT molecules from the cytoplasm. The STAT molecules are then phosphorylated (pSTAT) and form homo- or heterodimers that translocate to the nucleus where they regulate gene transcription [4]. STAT1 GOF patients show higher pSTAT1 levels after stimulation with activating cytokines (mainly interferons), which represents the molecular hallmark of the disease [1, 2]. Whether this is the result of altered dephosphorylation dynamics, prolonged binding of STAT1, increased availability of total STAT1 molecules, or other mechanisms remains to be elucidated [1, 2, 5].

From a clinical perspective, the phenotype of STAT1 GOF patients is broadly heterogenous. The most common symptom is early-onset chronic mucocutaneous candidiasis (CMC). However, (myco-) bacterial, viral, and fungal infections, (multiorgan) autoimmunity or autoinflammation, vascular malformations, and malignancies have also been reported [6]. The management of these complex patients is therefore challenging and often requires a balanced use of antimicrobial and immunosuppressive therapies. Hematopoietic stem cell transplantation (HSCT) is a potential curative procedure but graft failure as well as secondary graft rejection is common and resulted in a 40% overall survival rate only [7].

In recent years, case reports have described JAK inhibition as an effective targeted treatment option for STAT1 GOF patients [3, 8–12]. JAK inhibitors (JAKinibs) are small molecules interfering with the process of cytokine-dependent JAK activation. In patients with STAT1 GOF, ruxolitinib®, as well as baricitinib®, have been used [13, 14]. However, the clinical experience with these drugs in the field of inborn errors of immunity (IEI) is still limited and important questions including indications, dosing, and monitoring remain, especially in pediatric patients.

Here, we present the experience with JAK inhibition in 10 pediatric STAT1 GOF patients under the care of six IEI reference centers. We provide detailed information regarding indications, dosing regimens, side effects, and complications as well as the clinical effects on the most relevant disease manifestations. In addition, we reviewed all previously published pediatric STAT1 GOF cases treated with JAKinibs and compared main characteristics with our cohort, when applicable.

## Methods

### Patients and Study Design

Pediatric patients (age < 18 years at treatment initiation) with functionally confirmed or previously described STAT1 GOF mutations receiving JAKinibs for a minimum of two consecutive months were recruited from six IEI reference centers. The protocol of this study was reviewed and approved by the local ethics committees of the participating centers. Informed consents were obtained from study participants and/or their legal guardians according to the requirements of the local ethics committees.

### Data Collection

A questionnaire (available on reasonable request) designed to retrospectively collect demographic, molecular, and clinical data was prepared and distributed. Additionally, the responsible physicians were contacted to verify and discuss the extracted data for each patient.

### Genetic Analysis and Functional Variant Validation

All patients were tested at their corresponding institutions. Sanger sequencing was performed for all patients and novel variants were functionally validated by means of STAT1 phosphorylation assays as previously described [1, 2].

### Response Evaluation

For our cohort, the attending physicians were asked to categorize the clinical response of their patients before and after starting JAK inhibition in the following categories: (1) complete response, (2) partial response, (3) no response, (4) manifestation not present. Due to the limited data information extracted from the literature review, the treatment response of the published cases was categorized as follows: (1) resolution of symptoms or partial response, (2) manifestation present in the patient but response to JAKinib not specified, (3) transitory response, (4) no response, (5) manifestation not present.

### Immune Deficiency and Dysregulation Activity (IDDA) Score

The IDDA score is a promising tool to assess disease activity and burden in the setting of immune deregulatory diseases [15, 16]. It allows for intraindividual, longitudinal monitoring by using a number of relevant clinical parameters. Items required to calculate the score were part of a questionnaire. The patients’ scores were calculated as previously described [15] by their attending physician before starting JAK inhibition (retrospectively) and at the last clinical follow-up.

### Literature Review

STAT1 GOF patients less than 18 years old treated with JAK inhibitors were identified via a systematic literature search in EMBASE and PUBMED using the following search terms: primary immunodeficiency disease, inborn errors of immunity, pediatrics, children, JAK-STAT pathway, chronic mucocutaneous candidiasis, STAT1 GOF, JAK inhibitors, STAT1, gain of function, JAKinib, JAK inhibitor, ruxolitinib and baricitinib. All articles and references were screened for other eligible publications. To avoid case duplications, those patients mentioned in more than one publication were identified and relevant data was extracted from all corresponding publications (Table S-1).

### Statistical Analysis

Variables were described as percentages or median values with ranges (min–max), respectively. Normality for quantitative variables was evaluated using the Shapiro–Wilk test. For inferential statistics, the Wilcoxon test was applied. A *p*-value lower than 0.05 was considered statistically significant.

## Results

### Baseline Characteristics and Disease Manifestations Before Starting JAK Inhibition

Ten patients were included in our cohort (Table 1). Of note, patient 9 (P9) was treated with ruxolitinib during two time periods. Whilst the first episode has been previously published [11], we here provide extended data on the second treatment course. The baseline characteristics of the cohort are presented in Table 1 and a detailed description for each patient is given in Table S-1 and Table S-2.

**Table 1:** Summary of baseline characteristics of our cohort (*n*=10) and of previously described pediatric STAT1 GOF patients under Jakinib therapy (*n*=14)

	*n* = 10*	Literature review *n* = 14
Age (years) at time of study entry: mean	8.5 y (3y–18y)	10y (7 m–17y)
Gender (female)	8/10	7/14
Age (months) at symptom onset	6 (1–48)	4 (0.5–10)**
Mutations localization		
Coiled coil domain	4/10	2/14
DNA-binding domain	5/10	8/14
Linker domain	0/10	2/14
SH2 domain	1/10	1/14
Tail segment domain	0/10	1/14
Infections prior to JAK inhibitor	10/10	14/14
Viral	7/10	5/14
Fungal	10/10	13/14
Bacterial	8/10	11/14
Only Chronic mucocutaneous candidiasis	2/10	0/14
Immune dysregulatory symptoms	10/10	12/14
Cytopenia	3/10	9/14
Enteropathy	2/10	8/14
Autoimmune hepatitis	1/10	6/14
Endocrinopathy	0/10	4/14
Oral aphtha	8/10	2/14
Arthritis	1/10	0/14
Keratitis/episcleritis	4/10	1/14
Dermatitis/eczema	2/10	2/14
Fatigue	2/10	1/14
Alopecia	0/10	2/10
Lymphoproliferation	0/10	1/14
Pulmonary disease	6/10	5/14^**&**^
Bronchiectasis	6/10	5/14
Interstitial lung disease	0/10	0/14
Pulmonary hypertension	1/10	0/14
Failure to thrive	3/10	9/14
Vasculopathy	2/10	1/14
Heart	0	0/14
Central nervous system	2/2	1/14
Antibody deficiency***	5/10	3/14
Subclasses deficiency and hypo IgM and IgA	1	0
Subclasses deficiency and hypo IgA	1	0
Isolated low IgM	1	0
SPAD	1	0
Hypo IgG and SPAD	1	0
Isolated low IgG	0	1
Subclasses deficiency	0	1
Isolated low IgA	0	1
HSCT	2/10	4/14
Mortality	1/10	0/14
JAK inhibitor information		
Type of JAK inhibitor		
Ruxolitinib	9/10	14/14
Baricitinib	1/10	0/14
Starting dosage: median (range)		
Ruxolitinib	0.28 (0.2–0.6) mg/kg/day	20 (5–50) mg/day 11/14
Baricitinib	2 mg/day	5 (5–5) mg/m^2^/day 3/14
Maximum dosage: median (range)		
Ruxolitinib	0.6 (0.25–0.78) mg/kg/day	20 (5–50) mg/day (11/14)
Baricitinib	4 mg/day	10 (10–15) mg/m^2^/day (3/14)
Reason to start JAK inhibitors^&&^		
Uncontrolled immune dysregulation		
Oral aphtha	4/10	3/14
Keratitis/iritis	1/10	1/14
Enteropathy	1/10	6/14
Autoimmune hepatitis	1/10	2/14
Autoimmune cytopenia	1/10	4/14
Fatigue	0/10	1/14
Type I diabetes mellitus	0/10	2/14
Alopecia	0/10	1/14
Failure to thrive	0/10	2/14
Life-threatening infections	1/10	0/10
Recurrent bacterial infections	0/10	1/14
Chronic mucocutaneous candidiasis	6/10	4/14
Azole resistant	2/10	ND
Azole susceptible	4/10	ND
Vasculopathy progression	2/10	1/14
Lung disease progression/decline lung function	2/10	0/14
Bridge to HSCT	1/10	1/14
Median follow-up in months (range)	18 (2–42)	ND
Median IDDA score (range)		
Before treatment	15.99 (5.2–40)	
Under treatment	7.55 (3–14.1) *p* = 0.0078	ND
Side effects	4/10	
Infectious	1/4	4/14
Other	3/4	4/14
JAK inhibitor discontinued /stopped	3/10	ND

*CVID*, common variable immunodeficiency; *HSCT*, hematopoietic stem cell transplantation; *IDDA*, immune deficiency and dysregulation activity; *m*, months; *ND*, no detailed information was available for this variable; *SPAD*, specific polysaccharide antibody deficiency; *y*, year

^*^The results are expressed by median and range (min–max) and percentage if not stated otherwise

^**^Information available only from 4 patients

^***^Some patients presented more than one humoral defect

^&^One of these patients was stated to suffer from an unspecified chronic lung disease

^&&^Patients may have more than one reason to start ruxolitinib

The median age of disease onset and at study entry of our cohort was 6 months (range 1–48 months) and 8.5 years (3–18 years), respectively, with a predominance of female patients (8/10).

Infections were common and CMC was present in all patients, being the only infectious manifestation in two of them. Bacterial infections, mainly of the lower respiratory tract, were frequently reported; 6/10 patients developed bronchiectasis. At least one episode of symptomatic *herpesviridae* infection was observed in 6 out of 10 children prior to starting JAK inhibition.

All patients showed at least one autoimmune and/or autoinflammatory manifestation. Oral aphthae (8/10) were the most common feature, followed by scleritis/keratitis (4/10) and autoimmune cytopenia (3/10). Lymphoproliferation was not observed in our cohort. One patient suffered from pulmonary hypertension due to a chronic interstitial lung disease. Failure to thrive was noted in 3/10 patients, and in two patients, aneurysms of the central nervous system were identified.

Features of antibody deficiency were reported for 5/10 patients. Six of ten patients received immunoglobulin replacement treatment (IGRT).

A systematic literature review identified 14 additional pediatric STAT1 GOF patients under JAKinib therapy (see Table 1 and Table S-2. Median age was 10 years (7 months–17 years); 50% were female. Almost all children suffered infections, CMC (13/14) and bacterial (11/14) infections being most commonly reported. Autoimmune/autoinflammatory complications were often reported, with cytopenia being the most common (9/14), followed by enteropathy (8/14) and autoimmune hepatitis (6/14). One patient presented with lymphoproliferation and most showed failure to thrive (9/14).

### JAKinib Treatment Indications and Monitoring

In our cohort, 9 patients received ruxolitinib and one patient baricitinib. Dosing is detailed for each patient in Table S-1. The main reasons to start JAK inhibition were immune dysregulation (10/10), manifested as oral aphthae, keratitis, enteropathy and autoimmune hepatitis, followed by uncontrolled CMC (6/10). The baseline studies performed prior to treatment initiation and during the follow-up, as well as the monitoring frequency of the treatment, are detailed in Table 1, Figure S-1, Table S-1 and Table S-2. The median treatment follow-up time for our cohort was 18 months (2–42 months); with a total follow-up time of 197 months.

Although specific information was not available for all previously published cases the main reason to start treatment were autoimmune complications.

### JAKinib Treatment Responses

**Fig. 1:**
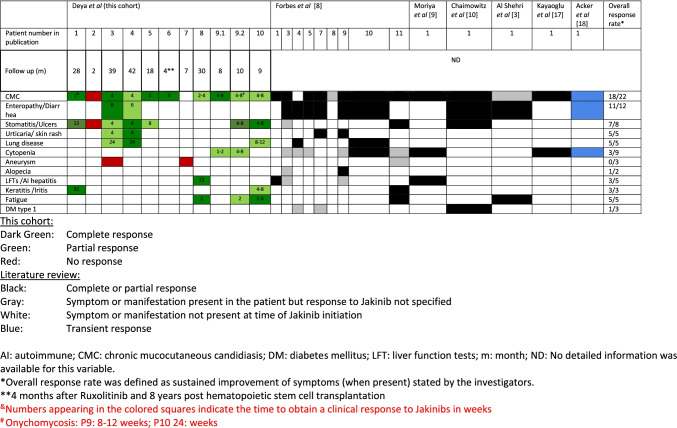
Response to JAK inhibitor treatment. AI, autoimmune; CMC, chronic mucocutaneous candidiasis; DM, diabetes mellitus; LFT, liver function tests; m, month; ND, no detailed information was available for this variable. *Overall response rate was defined as sustained improvement of symptoms (when present) stated by the investigators. **4 months after ruxolitinib and 8 years post hematopoietic stem cell transplantation. ^&^Numbers appearing in the colored squares indicate the time to obtain a clinical response to JAKinibs in weeks. ^#^Onychomycosis: P9: 8–12 weeks; P10: 24 weeks

An overview summarizing the treatment responses for each disease manifestation in our patients and previously published pediatric cases is shown in Fig. 1. Under JAKinib therapy, most clinical manifestations showed at least partial improvement except for P2, in whom CMC and stomatitis/aphthae persisted despite good treatment adherence. Time to response after treatment initiation appears to depend on the clinical manifestation. In our cohort, early responses were observed for cytopenia (1–2 weeks), CMC (1–8 weeks), dermatitis (2–4 weeks) and improvement of oral aphthae and enteropathy after 6–12 weeks of treatment. In contrast, keratitis, autoimmune hepatitis, or pulmonary hypertension required prolonged treatment (3–8 months) and cerebral aneurysms did not show any treatment responses during the time of follow up. In the reviewed cases from the literature, the variable “time to response” was not consistently reported (Figure S-1). In those cases where specific information was available, hemolytic autoimmune anemia (*n* = 1) responded after 1 month of treatment initiation [17] and enteropathy improved between 2 weeks and 2 months in three patients [8], whereas a singular more severe case required up to 12 months of therapy [18]. Resolution of diabetes mellitus was observed after 12 months of ruxolitinib treatment in 1 case [10].

**Fig. 2:**
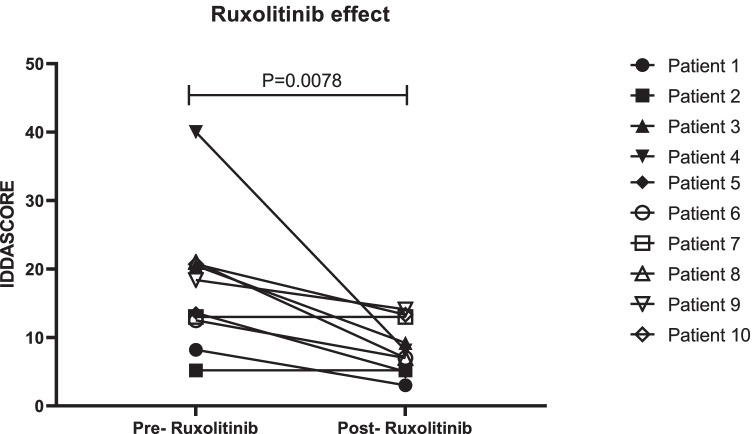
Effect of JAKinibs on Immune deficiency and dysregulation activity (IDDA) score

The IDDA score significantly decreased under ruxolitinib therapy (median pre: 15.99, median post: 7.55, *p* = 0.0078), whilst P2 (patient under baricitinib) and P7 did not improve at 2 and 7 months, respectively (Table 1, Fig. 2).

A summary of treatment responses reported for the previously published cases is shown in Fig. 1.Although detailed descriptions were not available for all patients and symptoms, most patients showed improvement under therapy for the most prevalent disease manifestations such as CMC, enteropathy, cytopenias, and lung disease.

### Prophylaxis and Adverse Events

Antibacterial (5/10) and antiviral (2/10) prophylaxis and immunoglobulin replacement therapy (5/10) were initiated prior to JAKinibs as part of the routine clinical management. In addition, antimicrobial prophylaxis was started after JAKinib initiation in two patients: in P1 due to recurrent bacterial lower respiratory tract infections and in P8 due to anticipated increased viral infection risk.

Bacterial infections (4 episodes during a 6-month period: 1 pneumonia and 3 episodes of upper respiratory tract infections with fever and acute reactants elevation), vertigo, sleep disturbances, and transitory liver enzyme elevation were attributed by the attending physicians to the JAKinib as probable but not proven side effects (Table S-1). Treatment was discontinued in 3/10 patients. P6 and P9 stopped ruxolitinib due to hepatotoxicity just before HSCT and loss of effect on CMC respectively. P2 discontinued baricitinib as no treatment benefit was observed after 2 months of therapy.

In the previously reported 14 patients, 8 presented adverse events potentially related to JAKinibs: 4/8 infections (2 cases *varicella zoster* virus, 1 *herpes simplex virus*, and 1 *cytomegalovirus* (CMV) and 4/8 suffered from other complications (one each from thrombocytopenia and neutropenia and two from pancreatitis).

### Ruxolitinib and HSCT

P6 (matched sibling donor) and P9 (matched unrelated donor) underwent HSCT. P5 is currently in the process of HSCT preparation. P6 was successfully transplanted and remains healthy and stable 4 years post-HSCT. P9 was treated twice with ruxolitinib therapy for 8 months prior to HSCT; however, the patient sadly deceased in the context of an uncontrollable thrombocytopenia and invasive aspergillosis 75 days post-HSCT (complete donor chimerism, no signs of graft-versus-host disease).

In three of the previously published 14 patients, ruxolitinib was stopped prior to HSCT. In this setting, HSCT was successful in all reported cases.

## Discussion

To the best of our knowledge, this is the most extensive pediatric case series describing patients with STAT1 GOF mutations under JAKinib therapy to date. Our study provides a detailed description of the clinical experience with this treatment approach in children and highlights the heterogeneity in terms of indications, dosing schedules, and follow-up practices.

### Disease Manifestations

Infections were common in our cohort, correlating well with previous reports [3, 8–10, 17, 18, 12]. Before starting JAKinibs, most infections had been controlled; only P3 suffered from CMV stomatitis. The previously published cases showed overall a more severe phenotype, with higher prevalence and severity of autoimmune manifestations and failure to thrive (Table 1, Table S-2). In addition, these patients had also received other immunosuppressive drugs. Thus, in most of them, JAKinibs were not used as “first-line” therapy. In our series, JAKinibs were initiated at earlier disease stages, possibly reflecting the positive experiences reported in the previous studies [3, 8–10, 17, 18, 12].

### Indications

Reasons to start JAKinibs stated by the attending physicians of our cohort were similar to previous reports [3, 8–10, 17, 18]. Beyond CMC, these included refractory autoimmune complications, progressive vasculopathy, and lung disease (Table 1, Table S1). Furthermore, one patient received ruxolitinib for 4 months as a bridge to a subsequent HSCT procedure [8, Table S-2].

### Treatment, Dosing, and Treatment Response

In the setting of IEI, the appropriate dosing and interval for JAKinibs remain to be established, as experience with these small molecule inhibitors in the pediatric age is very limited. Whilst the European Medicine Agency (EMA) has not yet approved ruxolitinib in children [19], the Food and Drug Administration (FDA) indicates their use for steroid-refractory acute graft-versus-host disease (GVHD) in children older than 12 years of age in 2019 (recommended dose 5 mg every 12 h) [20]. Of note, 50mg/m^2^/day has been indicated to be the maximum well-tolerated dose in children [21]. Based on serial drug level determination and functional assays, 8-h dose intervals have been recently suggested in a child with STAT3 GOF mutation associated with immune dysregulation (type 1 diabetes mellitus and interstitial lung disease). Interestingly, the dose needed and tolerated in this case report was high (2.2 mg/kg/day), being more than twice the dose compared to previous reports [8, 22] and those used in our own cohort (see Table S-1 and S-2).

In our patients, ruxolitinib was used in 9/10 and barcitinib in 1/10 children, respectively. The attending physicians preferred ruxolitinib, given the larger literary experience in STAT1 GOF setting. We provide detailed dosing information (Table 1 and Table S-1) for our patients, including the starting and maximum doses. Our starting (0.28 mg/kg/day vs 0.8 mg/kg/day) and maximum (0.6 mg/kg/day vs 1.05 mg/kg/day) doses were lower than previously reported [3, 8–10, 17]. However, the absence of homogenous protocols (and dosing reported diversely as mg/kg/day, mg/day, and/or m^2^/day) in the literature limits conclusive comparisons and should be unified in future studies. Dose adjustments in our cohort were performed mainly according to the clinical effect and absence of adverse events and in three cases supported by functional analysis using pSTAT1 stimulation assays (P3, P4, P9).

Collectively, CMC was the most prevalent disease manifestation (*n* = 23) and JAKinib treatment was effective in almost all patients (overall response rate 20/22, Fig. 1) within 2–8 weeks of treatment. Contrastingly, Acker et al. recently described a patient with only transient responses to JAKinib, administered for CMC, enteropathy, and cytopenia [18]. Importantly, in our cohort, the only patient receiving baricitinib did not show clinical improvement resulting in its discontinuation and switch to ruxolitinib.

In the absence of controlled prospective data, we suggest starting pediatric patients on 0.3–0.5 mg/kg/day of ruxolitinib twice per day and then progressively increasing the dose by 0.1–0.2 mg/kg/day every 2–4 weeks until achieving the expected clinical effect or occurrence of relevant side effects keeping in mind the suggested maximum dose of 50 mg/m^2^/day by Loh et al. [21].

For the clinician, the patients, and family, it is important to know how long it takes to achieve a JAKinib treatment response. In our cohort, the cytopenias and CMC responded rather promptly (1–8 weeks), whereas others, such as keratitis and autoimmune hepatitis, required prolonged treatment courses (4–8 months). No improvement or worsening of cerebral aneurysms was observed in two patients. Unfortunately, the information available in the literature regarding treatment responses is often unspecific and incomplete. Where such data were provided, the time to response was similar to what was observed in our cohort requiring several weeks of therapy to achieve improvement (Table S-1).

Despite the combined data presented here, the number of pediatric STAT1 GOF patients treated with JAKinibs is still small. Furthermore, it is likely that the time to response might vary depending on the organ involved, severity and duration of the disease, and JAKinib dosage. Therefore, larger, detailed, and prospective patient cohorts will need to address these aspects more consistently.

Baricitinib, a potent JAK1/JAK2 inhibitor, has shown good tolerability in rheumatologic diseases and other monogenic interferonopathies [23, 24]. To date, one case report indicated efficacy in an adult patient with STAT1 GOF suffering from recurrent aphthae, as well as oral and esophageal CMC [25]. Contrastingly, in our cohort, P2 failed to show any improvement after 2 months of treatment with 4mg/day.  However, upon, upon switching to ruxolitinib, a fast, complete, and sustained remission of CMC and partial remission of aphthae after 3 months of treatment were observed. Whether baricitinib is inferior or not in the control of the disease manifestations in STAT1 GOF compared to ruxolitinib remains to be determined.

### Assessing Disease Activity Using Immune Deficiency and Dysregulation Activity (IDDA) Score

The IDDA score is a promising tool to assess disease activity and burden in the setting of immune dysregulatory diseases [15, 16]. It allows for intraindividual, longitudinal monitoring by using a number of relevant clinical parameters and has been added as a voluntary option to the European Society for Immunodeficiencies (ESID) registry [26]. We applied the score for the first time to patients with STAT1 GOF obtaining lower numbers (15.99) when compared to those reported for lipopolysaccharide (LPS)-responsive and beige-like anchor protein (LRBA)-deficient patients proceeding to transplant (32.9) or those remaining under conventional immunosuppressive therapy (20.8) (Table 1, [15]). A significant reduction in the IDDA score was observed after initiation of JAKinib therapy for all patients with initial IDDA score > 10, suggesting a substantial decline in the disease activity after JAKinib introduction (Fig. 2).

### Adverse Events and Monitoring

Overall, the occurrence of adverse events potentially related to JAK inhibition was rare in our cohort. In fact, only one patient experienced an increased frequency of bacterial infections. Contrastingly, the reports in the literature for STAT1 GOF on JAKinib mention higher rates of urinary infections [27, 28] and other less frequent infectious complications, such as herpes virus reactivation [28], tuberculosis and/or other atypical mycobacterial infections [27–29], *JC virus* (four fatal cases) [30–33], *Pneumocystis jirovecii* [34], hepatitis B [35, 36], and toxoplasmosis [37]. This discrepancy might be attributed to an earlier introduction of JAKinibs in our cohort compared to their predominant use as a rescue strategy following the failure of other immunosuppressive regimens in the previously reported cases [3, 8, 10, 17, 18].

Although no published guidelines exist, we observed a surprisingly consistent approach chosen by the individual participating centers in terms of investigations performed prior to and during JAKinib therapy (Fig. S1, Table S1). These parameters most likely reflect concerns based on the published experience with JAKinibs in other scenarios, such as myelofibrosis, arthritis, and graft-versus-host disease (GVHD), as well as STAT1 GOF cases [8, 27, 28], FDA and EMA recommendations [19, 20]. They include screening for infectious complications and monitoring for organ toxicity. In the absence of an easy-to-perform assay to determine ruxolitinib serum levels and the lack of the well-defined correlation between drug levels and clinical response, other biomarkers have been explored to monitor the drug effect/clinical response, such as phosphorylated STAT1 levels (pSTAT1) and IL17 production in T lymphocytes. Whilst some studies suggest a correlation between normalization of these markers [3, 11, 38], others reported a clear discrepancy [5]. This might be due to differences in timing of sampling, sample preparation, and assay protocols. In future studies, harmonized treatment and monitoring protocols are needed to consistently evaluate the role of these and other biomarkers in patients with IEI under JAKinib therapy.

In our cohort, drug levels were not performed. All participating centers stated an overall interest to perform JAKinibs level testing but did not have test availability at their institutions.

Importantly, none of the patients described here experienced severe adverse events such as thromboembolism or pulmonary hypertension. Interestingly, one patient (P4), who was started on ruxolitinib despite suffering from pulmonary hypertension, showed a marked improvement allowing the reduction of chronic medication for pulmonary hypertension, as well as the suspension of long-term oxygen supplementation.

Our recommendation prior to starting the JAK inhibition in pediatric patients with STAT1 GOF is to obtain a complete medical history, aiming to identify previous, active, or chronic infections and potential underlying organ damage. We also suggest applying early and extensive diagnostic and therapeutic strategies when suspecting viral, bacterial, and/or fungal infections including blood, urine, stool, aspirate samples, and biopsies from affected tissues/organs, if indicated, to minimize the risk of severe and preventable infectious complications.

In the specific setting of JAK inhibition in (pediatric) STAT1 GOF patients, the role of primary or secondary antimicrobial, antiviral, and antifungal prophylaxis remains to be established. Most authors suggest antimicrobial prophylaxis in patients with recurrent (respiratory) infections [39]. Systematic prevention of herpes virus infections is more controversial but should be considered in those patients with a history of systemic infection and severe lymphopenia as well as a history of long-term immunosuppression. In our cohort, immunoglobulin replacement therapy and antimicrobial and antiviral prophylaxis were prescribed according to the initial immunological workup and were not part of a specific strategy to prevent infections under JAKinib therapy.

## Conclusions

We provide a comprehensive overview of the spectrum of pediatric STAT1 GOF patients that have been treated with JAK inhibitors to date, thereby highlighting the heterogeneity in terms of treatment indication, dosing, and monitoring. Based on our experience and previously published reports, we have stated recommendations regarding dosing, monitoring, and follow-up to help guide the attending clinicians. Application of a standardized methodology aimed to systematically assess the JAKinib indications, role of biomarkers, and drug level determination as well as clinical responses is needed and should be included in future studies. In this regard, the European Society for Immunodeficiency (ESID) and European Society for Blood and Marrow Transplantation (EBMT) have recently launched a multicentric retrospective study on JAKinib treatment in patients with inborn errors of the JAK/STAT pathways [40].

## Supplementary Information

Below is the link to the electronic supplementary material.

Supplementary file1 (DOCX 1.36 MB)

## Data Availability

The datasets generated during and analyzed during the current study are available from the corresponding author on reasonable request.
