# Supplementary material for: Impact of JAK Inhibitors in Pediatric Patients with STAT1 Gain of Function (GOF) Mutations—10 Children and Review of the Literature
Source: J Clin Immunol. 2022 Apr 29;42(5):1071–82. doi: 10.1007/s10875-022-01257-x (PMC9402491; doi:10.1007/s10875-022-01257-x)
Supplement: Supplementary file 1 — Supplementary file1 (DOCX 1.36 MB) [file 10875_2022_1257_MOESM1_ESM.docx]

**Supplementary material**

**Table S-1:** **Clinical manifestations, treatment monitoring and outcomes of pediatric STAT1 GOF patients**

|  | **Patient 1** | **Patient 2** | **Patient 3** | **Patient 4** | **Patient 5** | **Patient 6** | **Patient 7** | **Patient 8** | **Patient 9** |  | **Patient 10** |
| --- | --- | --- | --- | --- | --- | --- | --- | --- | --- | --- | --- |
|  |  |  |  |  |  |  |  |  | 1^st^ episode | 2^nd^ episode |  |
| **Previously referenced** | No | No | No | No | No | No | No | No | Yes [11] | No | No |
| **Age at diagnosis (years)/Sex** | 4y//F | 15y/F | 10y/F | 7y/F | 9y/F | 5y/F | 8y/M | 3y/F | 12y/M | 15y/M | 18y/F |
| **Age at onset of clinical symptoms (months)** | 1m | 48m | 6m | 6m | 12m | 9m | 3m | 6m | 2m | 2m | 6m |
| **Mutation and protein change** | c.866A>G  Tyr289Cys | c.894A>C p.Lys298Asn | c.1053G>T p.L351V | c.976C>T  p.Pro326Ser | c.A862C  p.T288P | c.1030A>G  p.Lys344Glu | c.1972A>C  p.N658H | c.G821A  p.R274Q | c.617T>C  p.L206P | c.617T>C  p.L206P | c.1169T>C  p.M390T |
| **Infection** | Recurrent herpetic stomatitis  Bacterial pneumonia (x2)  Periorbital cellulitis  Adenovirus Gastroenteritis (x2)  Recurrent oral CMC | Recurrent oral CMC | Recurrent skin abscesses with scaring  Recurrent LRTI (*H. influenzae*, *S. aureus*)  CMC  Persistent oropharyngeal HSV and CMV  Plantear verrugae  COVID19 | Recurrent bacterial LRTI  Recurrent gastroenteritis  Azole resistant CMC (mouth, Esophagus) | CMC  - Mouth  - Esophagus  - Genital area  Recurrent LRTI  Pneumonia  Severe S. *pyogenes* cervical abscess  CMV infection (mild, IgG positive) | CMC  - Mouth  - Esophagus  - Nail  Recurrent LRTI  Pneumonia  Mastoiditis  CMV infection | Recurrent bacterial and viral LRTI  Pneumonia  Paronychia (S aureus)  Esophagitis (CMV)  Gastroenteritis (Campylobacter coli)  CMC  Epiglottitis (*C. albicans*) | CMC  -Mouth  -Skin  -Nail | CMC (*C. albicans*, R, skin, mouth, nails)  Recurrent bacterial RTI (*H. infl, S. pneumoniae, S. aureus*)  Recurrent otitis (7x in 1^st^ year of life)  Unspecified viral infections | CMC (*C. albicans*, R, skin, mouth, esophagus, nails)  Hepatolienal candidiasis  Recurrent bacterial RTI (*H. infl, S. pneumoniea, S. aureus*, MRSA)  *Pneumocystis* jiroveci  Adenoviral pneumonia  Unspecified viral LRTI | CMC (*C. albicans*, R, mouth, skin esophagus, nails, esophagus,)  Recurrent bacterial RTI (*H. infl, S. pneumoniae, S. aureus*)  Recurrent viral LRTI  Recurrent VZV  Influenza  COVID19 |
| **Antibody deficiency** | Yes  Hypo IgM | No | Yes  Reduced vaccine responses | Yes  Low IgG  Reduced vaccine responses | No | No | Hypergammaglobulinemia (low IgG2 and IgA) | No | IgG subclass deficiency, low IgM | IgG subclass deficiency, low IgM and IgA | No |
| **Lymphoproliferation** | No | No | No | No | No | No | No | No | No | No | No |
| **AI cytopenia** | No | No | Lymphopenia | Lymphopenia | No | No | No. Low CD4 counts. | No | Lymphopenia | Anemia  Neutropenia  Lymphopenia | No |
| **Enteropathy** | No | No | Yes  Recurrent abdominal pain  and prolonged diarrhea | Villous atrophy | No | No | No | No | No | No | No |
| **AI hepatitis** | No | No | No | No | No | No | No | Yes | No | No | No |
| **Growth Failure** | No | No | No | FTT | No | Yes | No | No | Yes | Yes | No |
| **Pulmonary Disease**  **(Interstitial LD,**  **Bronchiectasis)** | Yes,  Bronchiectasis | No | Bronchiectasis | Recurrent wheezing  Bronchiectasis  Secondary pulmonary hypertension | Bronchiectasis | Bronchiectasis | No | No | No | No | Bronchiectasis |
| **Endocrinopathies** | No | No | No | No | No | No | No | No | No | No | No |
| **Vasculopathy** | No | No | Intracranial aneurysms (previous bleeding) | No | No | No | Yes. Dolichoectasia of the left vertebral and basilar artery. | No | No | No | No |
| **Arthritis** | No | No | No | No | No | Polyarthritis (hands) | No | No | No | No | No |
| **Other** | Recurrent oral aphthae  and keratitis | Recurrent oral aphthae | Recurrent oral aphthae | T21  Mild aphthous stomatitis  Mild-moderate atopic dermatitis  GE reflux | Perianal ulcers  Recurrent blepharitis | Episcleritis  Aphthous stomatitis | Recurrent aphthous stomatitis  Multiple fractures (5x)  Low bone density score  Mild eczema | Recurrent oral aphthae | No | Multiple esophageal strictures  Fatigue | Keratitis  Aphthous stomatitis  Fatigue  Lung function deterioration |
| **Active infections when starting Jakinibs (other than CMC)** | No | No | CMV stomatitis | No | No | No | No | No | No | No | No |
| **Previous Treatments / /Prophylaxis** | Colchicine  Tacrolimus (topical)  IRT (sc)  Fluconazole (FCZ)  Acyclovir | Hydroxychloroquine  FCZ | Acyclovir  Repeated course of empiric antibiotics for LRTI  FCZ  IRT (IV)  TMP-SMX | FCZ, Micafungin  Nystatin (topical)  Various antibiotic courses  Corticosteroid pulses  Sildenafil  IRT  TMP-SMX | Colchicine  Fluconazole | Topical Amphotericine B  Foscarnet | FCZ  TMP-SMX | FCZ/  Terbinafine  Cyclosporine/  Mycophenolate/  Azathioprine  G-CSF | FCZ or Itraconazole (oral)  TMP-SMX (prophylaxis)  IRT (sc) | Itraconazole (oral)  Posaconazole (IV. and oral)  Caspofungin/micafungin (IV)  Topical Amphotericine B  G-CSF  IRT (sc) | FCZ or Itraconazole (oral) or  Posaconazole (oral) or  Voriconazole (oral and IV)  Amphotericin B (topical)  TMP-SMX (prophylaxis)  Azithromycin (prophylaxis)  Topical steroids (Keratitis)  IRT (sc) |
| **Treatments started because of Jakinibs (e.g. prophylaxis)** | TMP-SMX | None | None | None | None | None | None | Acyclovir | None | none | None |
| **Medication during Jakinib therapy** | IRT (sc)  TMP-SMX  Acyclovir | None | IRT (sc)  TMP-SMX | IRT (sc)  TMP-SMX | Azithromycin | Topical Amphotericine B  Foscarnet | TMP-SMX | Acyclovir  Omeprazole  Terbinafine | Itraconazole (oral)  TMP-SMX (prophylaxis)  IRT (sc) | Posaconazole (oral)  Topical Amphotericine B  IRT (sc) | Posaconazole (oral) Amphotericin B (topical)  Azithromycin (prophylaxis – allergy to TMP/SMX)  IRT (sc) |
| **Reason for initiating Jakinibs** | Recurrent keratitis and aphtae | Recurrent aphthae despite hydroxiclorochine | STAT1 GOF diagnosis  Chronic fungal infection,  enteropathy,  aphthous ulcers,  progression of cerebral aneurysm | STAT1 GOF diagnosis  Azole resistant CMC  Recurrent infections  Severe progressive lung disease | STAT1 GOF diagnosis  Aphthae + chronic inflammation + severe life-threatening infection +  More local experience with Ruxolitinib than with other Jakinibs | STAT1 GOF diagnosis  Bridge to transplant | STAT1 GOF diagnosis  Progression of cerebral aneurysms | STAT1 GOF diagnosis  Chronic and severe fungal infection.  Autoimmune hepatitis | Refractory, persistent and azole resistant CMC  Previous experience with Ruxolitinib in hematologic diseases | Hepatolienal candidiasis  Refractory persistent cytopenias  CMC | Progressive lung disease  Recurrent azole resistant CMC  Previous experience with Ruxolitinib (pt9) |
| **Jakinib prescribed** | Ruxolitinib | Baricitinib | Ruxolitinib | Ruxolitinib | Ruxolitinib | Ruxolitinib | Ruxolitinib | Ruxolitinib | Ruxolitinib | Ruxolitinib | Ruxolitinib |
| **Dose (mg/kg/d) and frequency** | Start 0.2 mg/kg/d (BID)  Current 0.7 mg/kg/d (BID) | Start 2 mg/day (QD)  Current 4 mg/day (QD) | Start: 0.4mg/kg/d (BID) Current 0.5mg/kg/d (BID) | Initial 0.2mg/kg/d (BID), Current 0.25mg/kg/d (BID) | 0.35mg/kg/day BID  Current 0.78 mg/kg/d T.I.D | 0.28mg/kg/d (BID) | Initial 0.2mg/kg/d (BID)  Increased to 0.4mg/kg/d (BID) at 2 weeks  Increased to 0.6mg/kg/d (BID) at 4 weeks (current) | 0.6mg/kg/d (BID) | 0.3 mg/kg/d (BID), Increased to 0.7 mg/kg/d (BID) | 0.4-0.5 mg/kg/d (BID) | 0.2mg/kg/d (BID)  Increased to 0.4mg/kg/d (BID) (current) |
| **Weaning of immunosuppressive or anti-infective therapy** | Fluconazole  Colchicine  Tacrolimus | FCZ  Hydroxychloroquine | Stopped FCZ  Stopped Acyclovir | Stopped FCZ  Stopped Sildenafil  Stopped 02 suppl  Stopped nutritional support | No | No | FCZ (to avoid interactions) | Stopped prednisolone and cyclosporine | No | No | Amphotericin B (topical) |
| **IDDA-Score pre-/post- Jakinibs** | 8.2/3.0 | 5.2/5.2 | 20.5/9.2 | 40/8.1 | 13.6/5 | 12.5/7 | 13/13 | 21/7 | 18.3/14.1 |  | 20.7/13.33 |
| **Non elective admissions before/ during Jakinibs (Number of days/100d of the year before starting therapy)** | None | None | 20/none | 30/none | 10/none | 10/NA (HSCT) | None/none | 8/none | 10/None | 15/NA (HSCT) | 7/2 |
| **Clinical monitoring of adverse events (frequency)** | Yes  Day 7, 15 and at 1 month  Since then every 3 months | Yes  Day 7, 15 and at 1 month  Since then every 3 months | Yes  Day 15, 30  Monthly during 2 months  Since then every 3 months | Yes  Day 15, 30  Monthly during 2 months  Since then every 3 months | Yes  Day 15, 30  Every 1-2 months | Yes  Every 2weeks at the beginning then hospitalized bridge to HSCT | Yes  0,2,4 and 12 weeks  Then 3 monthly | Yes  Monthly | Yes  Day 3,7,14,30 from initiation  Every 3 months | Yes  Day 3,7,14,30 from initiation  Every month | Yes  Day 3,7,14,30 from initiation  Every 3 months |
| **Rationale for dose adjustments (clinical response and /or functional studies)** | Clinical response | Clinical response | Both | Both | Clinical response; systemic inflammation | Clinical response | No adverse events and functional studies | No dose adjustments  No adverse events | Both | Both | Both |
| **Adverse events** | Recurrent bacterial infections:  - 1 pneumonia (no admission required)  - 1 clinical sepsis without positive blood culture (admission required. No positive culture. Empiric ceftriaxone)  - 2 febrile episodes without focus/positive cultures treated with antibiotics | None | Vertigo | Abnormal sleep pattern | None | Therapy stopped before HSCT due to raised liver enzymes | None | None | None  Clinical worsening when acutely stopping medication | None | None |
| **Action following adverse event** | Starting TMP-SMX as prophylaxis | N/A | Follow-up MRIs | Specialist evaluation | N/A | Stopping | N/A | N/A | N/A | N/A | N/A |
| **Follow-up period since start of Jakinibs** | 28 months | 2 month | 39 months | 42 months | 18 months | 4 months  8 y post HSCT | 7 months | 30 months | 8 months | 10 months | 9 months |
| **Reason for stopping Jakinibs** | N/A | None response | N/A |  | N/A | Hepatotoxicity/HSCT | N/A | N/A | Loss of effect on CMC | Discontinued shortly prior to HSCT | N/A |
| **Alive or Dead** | Alive | Alive | Alive | Alive | Alive | Alive | Alive | Alive | Alive  (died after HSCT, see phase 2) | Deceased post HSCT | Alive |
| **Current status** | Healthy and stable | Healthy and stable | Healthy and stable | Healthy and stable | Healthy and stable, HSCT planned | Healthy and stable | Healthy and stable | Healthy and stable | Stable | Dead | Improved and stable |
| **Cause of death** | N/A | N/A | N/A | N/A | N/A | N/A | N/A | N/A | N/A | Uncontrollable thrombocytopenia and invasive aspergillosis | N/A |

sc: subcutaneous, IRT: immunoglobulin replacement treatment, IDDA: immune deficiency and dysregulation activity, JAKinib: JAK Inhibitor, TMP-SMX: Trimethoprim-sulfamethoxazole, y: year; d: day; BID: two times per day, QD: once a day TID: three times per day, LSS: Lymphocyte subsets; CMC: chronic mucocutaneous candidiasis, CMV: cytomegalovirus, DM: diabetes mellitus, EBV: Epstein Barr virus, FCZ: Fluconazole, FTT: failure to thrive, HHV-6: Human Herpes virus – 6, HSV: Herpes Simplex virus, HSCT: hematopoietic stem cell transplant, LFT: liver function test, PFTs: pulmonary function tests, TPN: total parenteral nutrition, N/A: not applicable, M: male; F: female; LRTI: lower respiratory tract infection; T21: trisomy 21.

**Figure S-1**

1. **Baseline studies performed prior starting JAK inhibitor treatment**

|  | P1 | P2 | P3 | P4 | P5 | P6 | P7 | P8 | P9.1 | P9.2 | P10 |
| --- | --- | --- | --- | --- | --- | --- | --- | --- | --- | --- | --- |
| FBC |  |  |  |  |  |  |  |  |  |  |  |
| LFT |  |  |  |  |  |  |  |  |  |  |  |
| Lipid profile |  |  |  |  |  |  |  |  |  |  |  |
| Renal function |  |  |  |  |  |  |  |  |  |  |  |
| Viral loads |  |  |  |  |  |  |  |  |  |  |  |

FBT: Full blood test; LFT: liver function test

|  | Performed |
| --- | --- |
|  | Not performed |

1. Tests performed under JAK inhibitor treatment (monitoring)

|  | P1 | P2 | P3 | P4 | P5 | P6 | P7 | P8 | P9.1 | P9.2 | P10 |
| --- | --- | --- | --- | --- | --- | --- | --- | --- | --- | --- | --- |
| FBC |  |  |  |  |  |  |  |  |  |  |  |
| LFT |  |  |  |  |  |  |  |  |  |  |  |
| Lipid profile |  |  |  |  |  |  |  |  |  |  |  |
| Renal function |  |  |  |  |  |  |  |  |  |  |  |
| Viral loads |  |  |  |  |  |  |  |  |  |  |  |
| LSS |  |  |  |  |  |  |  |  |  |  |  |
| Drug levels |  |  |  |  |  |  |  |  |  |  |  |
| IGAM |  |  |  |  |  |  |  |  |  |  |  |
| Th17 |  |  |  |  |  |  |  |  |  |  |  |
| pSTAT1 assays |  |  |  |  |  |  |  |  |  |  |  |
| Clinical exploration/ follow-up |  |  |  |  |  |  |  |  |  |  |  |

|  | Performed |
| --- | --- |
|  | Not performed |

FBC: Full blood count; IGAM: immunoglobulins G, M and A; LFT: liver function test; LSS: lymphocytes subsets; pSTAT1: phosphorylated STAT1; Th17: IL17 producing T cells

**Table S-2**. **Literature review describing 14 pediatric patients with STAT1 GOF mutation treated with Ruxolitinib**

| **Authors/Journal** | **Study and Number of patients** | **Main Results** |
| --- | --- | --- |
| Forbes LR, *et al.*  J Allergy Clin Immunol  2018  **P4 same as P9 in the Vargas-Hernandez *et al*, J Allergy Clin Immunol. 2018  **P7 same as in Weinacht *et al*, J Allergy Clin Immunol. 2017. | 17 patients with STAT1 GOF (11/17) or STAT3 GOF (6/17)  **9 pediatric STAT1 GOF patients**  P1 10y/F (c.604A>G; p.M202V)  - Ruxolitinib 1.05mg/kg/day  P3 11.25y/F (c.917G>A; p.C324Y)  - Ruxolitinib 1-1.5mg/kg/day  P4** 11y/M (c.983A>G; p.H328R)  - Ruxolitinib 0.7-0.9mg/kg/day  P5 11.75y/M (c.1053G>T; p.L351F)  - Ruxolitinib 0.8-1.0mg/kg/day  P7 17y/M (c.1154C>T; p.T385M)  - Ruxolitinib 0.4mg/kg/day  P8 7.5y/F (c.1398C>G; p.S466R)  - Ruxolitinib 1.2mg/kg/day  P9 13m/F (c.1627T>C;p.C543R)  - Ruxolitinib 0.6-1.2mg/kg/day  P10 10y/F (c.1633G>A; p.E545K)  - Ruxolitinib 5-10mg/m^2^/day  P11 (12y/M (c.1825G>A; p.E609K)  - Ruxolitinib 5-10mg/m^2^/day | Indication:   - - Refractory Autoimmunity AND CMC: P3, P5, P7, P9   - Refractory autoimmune disease: P1, P4, P10, P11   - P8 received Ruxolitinib prior to HSCT   Clinical effect:   - - Good overall response in all patients. No deaths.   - CMC resolved in all cases   - Control of immune dysregulation symptoms: Diarrhea, cytopenias, ulcers, PFTs, alopecia   Molecular effect:   - - Not specified on an individual level for all patients   - P4 (data extracted from Vargas-Hernandez et al) pSTAT1 normalization, restoration of NK cell function   - P7 (data extracted from Weinacht *et al*.), pSTAT1 normalization, Th17 restoration after 12 months of therapy, IFNγ production in CD4+ cells and normalization of TfH (3-4 months)   Time to clinical improvement:   - - P3: Fungal infection improvement within 2 months   - P4: Decreased/normalized stool output within 1 month/3months   - P7: CMC and diarrhea within 3 months   - P9: Decreased/normalized stool output within 4 weeks   Side effects:   - - Cytopenias (3/9)   - Transient transaminases elevation (1/9)   - Mild pancreatitis (2/9)   - Infections: H zoster (P3, P11), Ruxolitinib was held, and antiviral medication started; Human metapneumovirus (P3). Infections cleared with supportive care along with JAKinib treatment.   - P3, P11: Ruxolitinib was held in the context of H zoster infection and not restarted in P11 due to parental choice, with relapse of symptoms (CMC, PJP)   Overall follow up:   - - Range 4-34 months (median 11 months). Not specified in all cases if on Ruxolitinib   Other:   - - P4 previously transplanted   - P8 received Ruxolitinib for 17 days as bridge to HSCT (stopped day -9) |
| Al Shehri T, *et al*.  J Clin Immunol 2019 | 1 patient: 10y/M  (c.2113G>C p.E705Q)  Ruxolitinib 33 mg/m^2^/day. | Indication:   - - Previous infection susceptibility had improved with antibiotics (azithromycin, fluconazole, valacyclovir and IRT)   - Continued with severe recurrent aphthous stomatitis   - Generalized fatigue   Clinical effect of Ruxolitinib (“shortly” after initiation):   - - Improvement of stomatitis and fatigue   Molecular effect:   - - STAT1 hyperphosphorylation normalized   - CXCL9, CXCL10, IRF1 reduced   - No effect on IL-17 or IL-22 production   Time to clinical improvement:   - - “shortly” after initiation   Side effects:   - - Herpes zoster   - LRTI requiring hospitalization and IV antibiotics   Treatment duration:   - - Not specified   Other:   - - HSCT (MUD) successfully performed   - At 15 months post HSCT symptom-free, 100% donor |
| Moriya K, *et al.*  Int J Hematol.  2020. | 1 patient: 3 y/M (c.1154C>T; p.T385M)  Ruxolitinib 5mg/m^2^/day | Indication:   - - Autoimmune manifestations (pancytopenia, AI hepatitis)   - Controlling side effects of prolonged systemic corticosteroids (growth impairment, glaucoma, hypertension)   - Only partial response to Rituximab   Clinical effect:   - - Complete resolution of CMC, autoimmune hepatitis   - Stopped corticosteroids   - No autoimmunity since start of therapy   Molecular effect:   - - Normalization of pSTAT1 (Western Blot, IFNg 30 min)   - Th17 (peripheral memory CD4+IL17A+) cells unchanged   - Normalization of IFNg in memory CD4+ cells   Time to clinical improvement:   - - Not specified   Side effects:   - - Mild CMV reactivation without complications.   Overall follow-up:   - - Not specified |
| Chaimowitz, *et al.*  N Engl Med.  2020. | 1 patient: 15y/M  (c.1154C>T, pT385M)  Ruxolitinib: 20mg/day | Indication:   - - Type I diabetes, chronic diarrhea, oral and rectal ulcers, CMC, sinopulmonary infections   Clinical effect:   - - Resolution of CMC and autoimmunity (including type 1 diabetes)   - Decreased rate of infections   Molecular effect:   - - Not specified   Time to clinical improvement:   - - “few” months after starting Ruxolitinib   - Gradual decrease in insulin requirements (stopped after 12 months of Ruxolitinib)   Side effects:   - - None stated in the case report   Treatment duration:   - - 27 months |
| Acker KP *et al.* J Pediatr Gastroenterol Nutr. 2020 | 1 patient 1y/F  (c.1627T > C, p.Cys543Arg)  Ruxolitinib 15mg/m^2^/dose BID | Indication:   - - Very early onset inflammatory bowel disease   - Failure to thrive   - Autoimmune cytopenia’s   - Anogenital candidiasis and abscesses   Clinical effect:   - - Improved diaper candidiasis   - Improved autoimmune cytopenia’s   - Enteral sufficiency (transient)   Molecular effect:   - - Not stated   Time to clinical improvement:   - - 5 weeks for candidiasis   - Not specified for the other features   Side effects:   - - Oral herpes simplex virus infection   Treatment duration:   - - 12 months   Other:   - - Endoscopic and histologic findings 6 months after initiation of Ruxolitinib did NOT show any improvement   - Clinical benefit described as transitory |
| Kayaoglu B *et al.*  J Clin Immunol.  2021 | 1 patient: 3.5 y/F  (c.1154C > T, T385M)  Ruxolitinib 10-20mg/m^2^/day  - dose adjusted according to pSTAT1 assay | Indication:   - - Recurrent bacterial pneumonia, recalcitrant oral and laryngeal candidiasis, fungal pneumonia, CMV   - Steroid dependent hemolytic anemia with persistent + coombs assay   Clinical effect:   - - Discontinuation of steroids   - Resolution of CMC   Molecular effect:   - - Decreased IFN-γ production   - No restoration of IL-17   - Downregulation of IFN-related genes   - Dysregulated T follicular helper normalized   Time to clinical improvement:   - - 1 month   Side effects:   - None   Treatment duration:   - 7 months stopped Day -1 of HSCT   Other:   - At 20 month post HSCT alive, well 100% donor |

AI: autoimmune, CBC: complete blood count, CMC: chronic mucocutaneous candidiasis, CMV: cytomegalovirus, F: Female, GOF: gain of function, HSCT: hematopoietic stem cell transplantation, IFNg: Interferon gamma, IRT: Immunoglobulin replacement therapy, LFTs: liver function tests, LRTI: lower respiratory tract infection, M: male, MUD: matched unrelated donor, PFTs: pulmonary function test, PJP: *pneumocystis jirovecii* pneumonia, pSTAT1: phosphorylated STAT1, TfH: T follicular helper cells, y: years

**Figure S-2. (A): Summary of the STAT1GOF mutations identified in pediatric patients. The following figure summarizes the different STAT1GOF mutations described so far. In blue, STAT1 GOF mutations identified previously in the literature (REF. 3, 8, 9, 10, 11, 16). In red, STAT1 GOF mutations identified in our cohort and described previously (Patients 1,3,5,6 by Toubiana *et al.^6^*; patients 8 and 9 by Bloomfield *et al*.^11^ and patients 3 and 10 by Vargas-Hernández ^et al.^ ^40^). In black, STAT1 GOF mutations identified in our cohort but not described previously in the literature. A number is included for each mutation allowing to identify the corresponding patient from the table S-1. (B) For the two new mutations described in our cohort, we provide an evaluation of the JAK/STAT1 pathway by flow cytometry. Histogram represents phospho-STAT1 geometric mean fluorescence intensity (gMFI). Left panel: expression of phospho-STAT1 (pSTAT1) in CD14 Patient 4 (P326S) and healthy control after IFN***γ* **stimulation. Right panel: expression of phospho-STAT1 (P-STAT1) in CD14 Patient 7 (N658H) and healthy control after IFN***γ* **stimulation. NS, non-stimulated; IFN***γ,* **stimulated with IFN***γ***; Pt4, patient 4; HC, healthy control.**


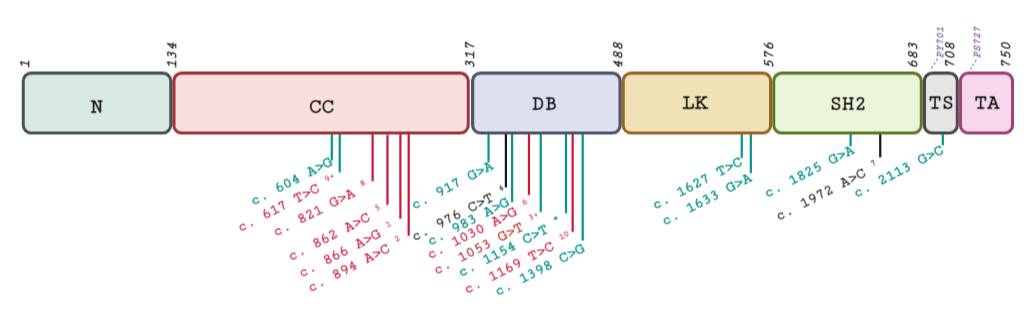


A)

CC, coiled-coil domain; DB, DNA-binding domain; LK, linker domain; N, N-terminal domain; SH2, SH2 domain; TS, tail segment; TA, transcriptional activation domain. * Three different cases found with the same mutation.

CC, coiled-coil domain; DB, DNA-binding domain; LK, linker domain; N, N-terminal domain; SH2, SH2 domain; TS, tail segment; TA, transcriptional activation

domain. * Three different cases found with the same mutation.


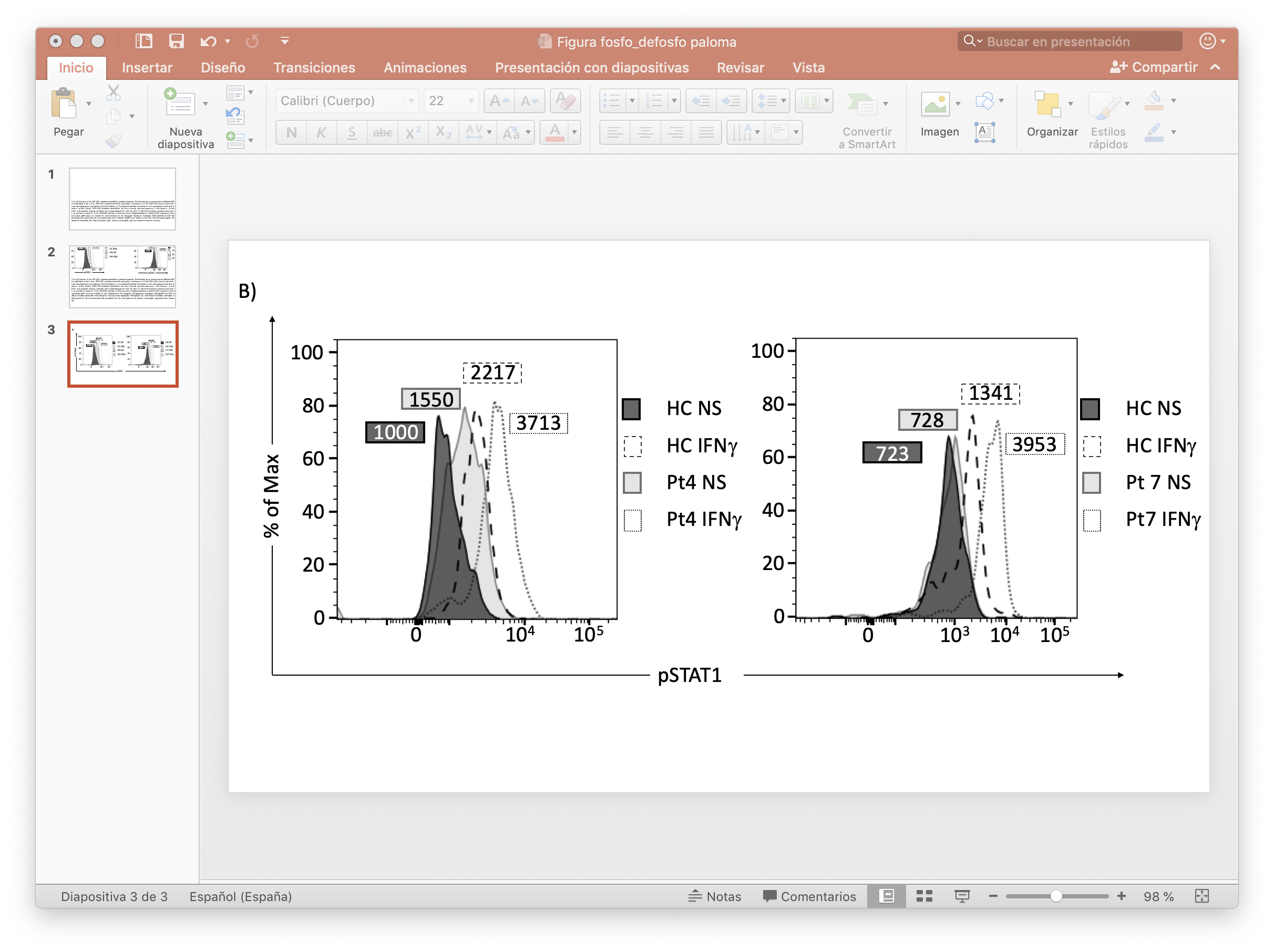

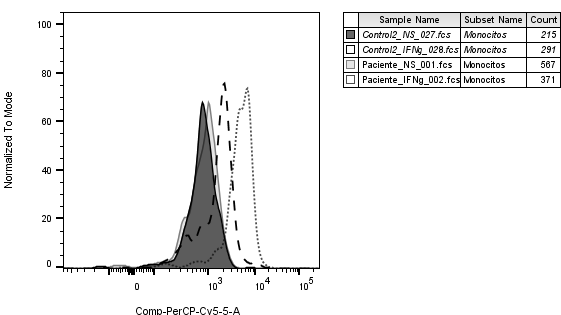


B-2)

HC NS

Pt 7 NS

HC IFNγ

Pt7 IFNγ
